# Unlocking the Potential of *Bacillus subtilis*: A Comprehensive Study on Mycotoxin Decontamination, Mechanistic Insights, and Efficacy Assessment in a Liquid Food Model

**DOI:** 10.3390/foods14030360

**Published:** 2025-01-22

**Authors:** Donato Greco, Vito D’Ascanio, Elisa Santovito, Mariagrazia Abbasciano, Laura Quintieri, Clarisse Techer, Giuseppina Avantaggiato

**Affiliations:** 1 Institute of Sciences of Food Production (ISPA), National Research Council (CNR), Via Amendola 122/O, 70126 Bari, Italy; donato.greco@cnr.it (D.G.); vito.dascanio@cnr.it (V.D.); elisa.santovito@cnr.it (E.S.); mariagrazia.abbasciano@ispa.cnr.it (M.A.); laura.quintieri@cnr.it (L.Q.); 2 Mixscience, 2/4 Avenue de Ker Lann, CS17228, CEDEX, 35172 Bruz, France; clarisse.techer@mixscience.eu

**Keywords:** *Bacillus subtilis*, mycotoxins, ZEA decontamination, liquid food model, mycotoxin metabolization

## Abstract

Mycotoxin detoxification by microorganisms offers a specific, economical, and environmentally sustainable alternative to physical/chemical methods. Three strains of *B. subtilis*, isolated from poultry farm environments and recognized by EFSA as safe in animal nutrition for all animal species, consumers, and the environment, were screened for their ability to remove mycotoxins. All of them demonstrated mycotoxin-dependent removal efficacy, being very effective against ZEA and its analogues (α- and β-ZOL, α- and β-ZAL, and ZAL) achieving up to 100% removal within 24 h under aerobic, anaerobic, and restrictive growth conditions with toxins as the sole carbon source. ZEA removal remained effective across a wide range of pH values (5–8), temperatures (20–40 °C), and at high toxin concentrations (up to 10 µg/mL). Additionally, up to 87% ZEA removal was achieved after 48 h of incubation (30 °C) of the strains in a contaminated liquid food model containing 1 µg/mL of the toxin. Mechanistic studies suggest that ZEA detoxification involves metabolic processes rather than physical adsorption or entrapment into bacterial cells. Enzymatic activities within the bacterial cells or associated with their cell walls likely play a role in the metabolization of the toxin. Interestingly, it has been observed that growth conditions and culture media can influence the metabolization and/or conjugation of the toxin, which can result in the production of various metabolites. Further investigation is needed to identify these metabolites and assess their safety.

## 1. Introduction

The presence of mycotoxins is a significant global challenge, detrimentally affecting agricultural productivity and compromising the quality and safety of agricultural products. This predicament leads to an increasing risk for animal and human health, as well as substantial financial losses for both food and feed chains [1,2,3]. In response to these challenges, several detoxification practices for detoxifying feed and food by mycotoxins have been proposed; however, most of them show limits for practical applications [4,5,6]. Biological methods represent a valuable approach as they are specific, efficient, and environment friendly [7,8]. Recently, scientists have focused their attention on the mycotoxin decontamination by microorganisms (bacteria and yeast) or enzymes which have been proposed to decontaminate foodstuffs from aflatoxin B1 (AFB1), deoxynivalenol (DON), zearalenone (ZEA), and fumonisin B1 (FB1) [7,8,9,10,11,12,13,14]. Biological-based methods for mycotoxin decontamination include mycotoxins’ degradation, transformation, and/or adsorption [15]. However, most microorganisms are not suitable for application in food or feed commodities. Issues such as the weak binding of mycotoxins to bacterial cell walls and their release during gastrointestinal digestion, the toxicity of breakdown products following mycotoxin degradation, and the potential unfavorable consequences of fermentation by non-native bacteria are all subjects of ongoing debate [16]. Beneficial probiotic bacteria such as lactic acid bacteria (LAB) and *Bacillus* (some of them recognized as GRAS), already known for their effect in enhancing animal performance [17], have been proposed for a wide variety of applications in the feed industry [18,19,20,21,22], including the decontamination of feedstuffs by mycotoxins [23,24,25,26,27]. *Bacillus* species are known for their role as mycotoxin detoxifiers by adsorption to the cell wall structure and biotransformation through different enzymatic activity exerted by enzymes such as carboxypeptidases, lactonases, and laccases [15,28,29,30]. Among *Bacillus* species, *B. subtilis*, a Gram-positive and spore-forming soil bacterium that is typically classified as an aerobe, is capable of growth within many environments even in the absence of oxygen [31,32]. It shows rapid growth and ease of cultivation, making it useful for various industrial applications [33,34,35]. *B. subtilis* has been proposed both as biocontrol agent against a variety of toxin-producing pathogenic fungi and as a microbial supplement to enhance intestinal function in animals [36,37]. As a probiotic, it is known to stimulate animal growth as well as to safeguard animal health, inhibiting the growth of pathogenic bacteria showing resistance under unfavorable conditions within simulated gut environments [38,39,40,41,42,43,44,45]. Due to these properties, the use of *B. subtilis* as feed additives has also been recently authorized by EFSA [46]. Several studies have highlighted the effectiveness of various strains of *B. subtilis* in decontaminating mycotoxins in vitro [7]. Farzaneh et al. [47] proved that the isolated *B. subtilis* strain UTBSP1 was able to degrade more than 90% of AFB1 in pistachio nuts. Similarly, Gao et al. [48] showed that the *B. subtilis* strain ANSB060, isolated from fish gut, showed a strong ability to detoxify aflatoxinB1 (82%), aflatoxin M1 (60%), and aflatoxin G1 (81%) after 72 h incubation in an LB medium *B. subtilis* ASAG 216, isolated from the intestine of a donkey detoxified LB medium from DON in a wide range of temperature and pH conditions [49]. Similarly, several strains of *B. subtilis* were effective in detoxifying ZEA in vitro. Ju et al. [50] found a 100% ZEA decontamination by cultured *B. subtilis* 168 in an LB medium for 48 h. Interestingly, this strain also removed more than 60% of ZEA in liquid food such as beer. The ZEA decontamination ability exerted by this strain was mainly dependent on adsorption of the toxin, followed by its degradation. Furthermore, Lei et al. [44] found that the *B. subtilis* ANSB01G isolated from normal broiler intestinal chyme degraded, under simulated intestinal tract conditions, more than 80% of ZEA in naturally contaminated maize and swine complete feed. In addition, this strain also decontaminated ZEA-contaminated dried distillers’ grains containing solubles (DDGS). It also showed antimicrobial activities against pathogenic bacteria and resistance to simulated gut environments; and it removed the estrogenic effects on growth performance, vulva size, relative weights of genital organs, serum hormone, and the reproductive organ histomorphology of pre-pubertal female piglets fed moldy corn naturally contaminated with ZEA. Despite many studies on the effectiveness of *B. subtilis* in decontaminating mycotoxins in vitro, there are several aspects that require further clarification [51]. The literature lacks information on the ability of *B. subtilis* strains to remove other mycotoxins including ZEA analogues which show estrogenic activity similar to or higher than ZEA [52,53]. It is well-understood that in vivo, ZEA is primarily converted to α- and β zearalenols (α- and β-ZOL) through the reduction of the ketone group to a hydroxyl group [54]. These metabolites show acute reproductive and genetic toxicity, with is in the order α-ZOL > ZEA > β-ZOL [55]. Additionally, the efficacy of *B. subtilis* strains in removing mycotoxins in anaerobic growth conditions has not been evaluated. This latest aspect could be of great relevance for various applications, such as the decontamination of ZEA-contaminated silage and other fermented food/feed models, or the inactivation of the mycotoxin in the digestive tract of animals. Lastly, there is a need to study the mechanism involved in the degradation processes as well as to identify the structure of degradation products and/or their residual toxicity [56].

In response to this demand, this research paper explores the potential of three strains of *B. subtilis* recognized as safe by the European Food Safety Authority (EFSA) in removing some of the major mycotoxins normally found in food and feedstuff, i.e., AFB1, DON, ZEA, FB1, ochratoxin A (OTA), and T-2 and HT-2 toxins. A special focus was dedicated to the efficacy of these strains in removing the toxin ZEA, and monitoring, at the same time, the formation of possible metabolites such as α- and β zearalenols (α- and β-ZOL), α- and β zearalanols (α- and β-ZAL) and zearalanol (ZAL) that may be generated by the decontamination process. The rationale for investigating these strains lies in the well-documented probiotic properties of *B. subtilis*, which extend beyond their traditional use in food supplementation. This research enabled a comprehensive analysis including different culture media, growth conditions (aerobic and anaerobic), incubation times, temperatures, and pH. The aim was not only to delineate the strains’ ability to remove ZEA (and its metabolites) but also to elucidate the underlying mechanisms governing this process. The mycotoxin removal activity was also explored in a liquid food model (LF). Typically, mycotoxins in LF can be derived from the contaminated raw materials used to prepare liquid formulations, as suggested by some authors [57,58,59]. As mycotoxin contamination poses substantial challenges to both crop yield and human health, this study aims to contribute valuable insights into the development of bio-based solutions, paving the way for a safer and more environmentally conscious approach, according to the European regulation [60,61].

## 2. Materials and Methods

### 2.1. Chemicals

All chemicals used were of analytical grade unless otherwise stated. All solvents (HPLC grade) were purchased from J.T. Baker (Deventer, The Netherlands). The water was of Milli-Q^®^ quality (Millipore, Bedford, MA, USA). Chemicals, where not otherwise specified, were from Sigma–Aldrich (Milan, Italy). Phosphate-Buffered Saline (PBS) tablets were purchased from VWR International (Milan, Italy). Pure standards in powder (purity > 99%) AFB1, FB1, T-2 and HT-2 toxins, ZEA and ZEA analogues (α- and β-ZOL, α- and β-ZAL, and ZAL) were supplied by Fermentek Ltd. (Jerusalem, Israel). OTA and DON were purchased from Sigma-Aldrich (Milan, Italy). Mycotoxin stock solutions (1 mg/mL) were prepared by dissolving solid commercial toxins in acetonitrile (HPLC grade). FB1 stock solution was prepared in acetonitrile/water (50:50). All mycotoxin stock solutions were stored in the dark at 4 °C. The Tryptic Soy Broth (TSB) and the minimal microbial growth medium (M9, Minimal Salts, 5X) were supplied by Biolife Italiana srl (Milan, Italy) and Sigma Aldrich srl (Milan, Italy), respectively. The sodium thioglycolate used for anaerobiosis was purchased by Sigma-Aldrich s.r.l. (Milan, Italy). AflaTest^TM^, FumoniTest^TM^, OchraTest^TM^, ZearalaTest^TM^ and DonTest^TM^ wide bore immunoaffinity columns were purchased by VICAM (Waters Corporation, Milford, MA, USA). T-2/HT-2 analysis was performed using EASI-EXTRACT^®^ T-2 and HT-2 immunoaffinity columns provided by r-Biopharm (Milan, Italy). The β-glucuronidase/arylsulfatase (30/60 U/mL) and the sulfatase (≥2000 U/mL) both from *Helix pomatia* were purchased by Sigma Aldrich srl (Milan, Italy). The β-glucuronidase was provided by Roche Diagnostics (Mannheim, Germany).

### 2.2. Bacterial Strains and Culture Conditions

*B. subtilis* A5, B7, C9 were provided by Mixscience (NOLIVADE, Château-Gontier-sur-Mayenne, France). These strains were originally isolated from the environment at poultry farms located in France and are deposited in the National Collection of Cultures of Microorganisms (CNCM)—Institute Pasteur (Paris, France) with the following accession numbers: CNCM I-4606 (A5), CNCM I-5043 (B7), CNCM I-4607 (C9). Stock cultures were stored at −80 °C in TSB containing 20% (*v*/*v*) of glycerol until required. Prior to use, strains were aseptically loop-inoculated from frozen stock cultures in sterile TSB (10 mL) and incubated overnight at 30 °C until OD_600nm_ of 1 (ca. 9 Log CFU/mL) was reached. A minimal medium (MM) was prepared according to Warner and Lolkema [62] and used for aerobic growth. When used for anaerobic testing, MM was supplemented by 0.2% of KNO3 (MMN) according to Glaser et al. [63]. To further promote microbial growth in anaerobic conditions, the MMN was supplemented with 2% (*w*/*v*) of glucose as carbon source (MMNG). Anoxic atmosphere was generated using Oxoid AnaeroGen 2.5 L sachets (Sigma Aldrich srl, Milan, Italy) placed into in sealed jars. The pH was adjusted as suggested by Gauvry et al. [64] by adding 1 M sodium hydroxide or 1 M hydrochloric acid sterilized on 0.22 µm filters. Microbial growth before and after the mycotoxin decontamination experiments (at 24 and 72 h) was assessed by plate counting on Tryptic Soy Agar (TSA), incubated at 30 °C for 24 h.

### 2.3. Mycotoxins Decontamination Tests

The efficacy of *B. subtilis* strains in removing AFB1, FB1, OTA, DON, T-2, HT-2, ZEA and zearalenone analogues (α- and β-ZOL, α- and β-ZAL, and ZAL) was assayed in both aerobic and anaerobic growth conditions. TSB and MMNG were used as growth media for aerobic and anaerobic decontamination tests, respectively. To perform the tests in aerobic conditions, 50 μL of the overnight *B. subtilis* cultures were diluted and inoculated in Sterilin™ test tube containing 5 mL of sterile TSB supplemented with 1 µg/mL of each mycotoxin (AFB1, FB1, OTA, DON, T-2, HT-2, ZEA and ZEA analogues). Thus, each strain was inoculated at ca. 6 Log CFU/mL and incubated in an orbital shaker set at 30 °C and 150 rpm over 24 and 72 h. After incubation, samples were centrifuged for 20 min at 14,000 rpm and 4 °C and the supernatants were analyzed by HPLC/UPLC methods as described in Section 2.4. Inoculated TSB without mycotoxins and sterile TSB containing mycotoxins were also included in the assays as negative and positive controls, respectively. The positive controls were used to assess the stability of mycotoxins in the different growth conditions. Mycotoxin decontamination tests were also performed using a pool of the three strains inoculated at 6 Log CFU/mL each. In addition, mycotoxins removal tests were performed in anoxic growth conditions by incubating the three *B. subtilis* strains for 72 h in a MMNG medium instead of TSB. Microbial loads were monitored after 0 and 72 h by plate counting onto TSA as described in Section 2.2. Unless otherwise stated, all tests were repeated in three independent experiments on different days.

### 2.4. LC Analysis of Residual Mycotoxins

The supernatants obtained by the mycotoxins decontamination tests in TSB required a clean-up step prior to LC analysis; no clean-up was necessary when mineral media (MM and MMNG) were used as growth media. Sample clean-up was performed by passing (one drop per second) each supernatant through immunoaffinity columns (IMA), previously anchored to a vacuum manifold (Visiprep™ SPE, Sigma Aldrich, Milan, Italy). Aliquots of 0.1 to 1 mL were used depending on the IMA. IMA columns were then washed by adding 5 mL of PBS followed by 5 mL of water. After the washing steps, AFB1, FB1, OTA, ZEA and analogues, DON, T-2, and HT-2 were eluted in 4 mL salinized amber vials using 2 mL of methanol. FB1 was eluted using 2 mL of methanol followed by 2 mL of water. Eluates were dried at 50 °C under an air stream (nitrogen was used for FB1) and the residues were re-dissolved in 0.25 mL of 20% methanol in water. The tubes were vortexed for a few minutes and injected into the LC systems. Chromatographic analysis of AFB1, FB1, OTA, DON, T2 and HT-2 was performed, following the protocol described by Greco et al. [65]. ZEA and its major metabolites were analyzed following the method described by Ragoubi et al. [29]. Both analytical methods fit the purpose of the study since they guaranteed high sensitivity, along with excellent selectivity, accuracy, and precision (RSDs < 6%). The limits of quantitation (LOQ) were 70 ng/mL for DON; 7 ng/mL for ZEA; 0.7 ng/ mL for OTA and AFB1; 50 ng/mL for FB1; 100 ng/mL for T-2 and HT-2 (S/N ratio = 10). These values (1–4 orders of magnitude lower than the toxin concentrations in the working solutions) ensured accurate LC measurements even when significant mycotoxin adsorption (>90%) took place.

### 2.5. ZEA Decontamination: Effect of Incubation Time, Temperature, pH, and Toxin Concentration

To find the optimal conditions for ZEA decontamination, the effects of incubation time, temperature, pH, and toxin concentration on ZEA decontamination activity by the strains A5, B7 and C9 were further assessed. Decontamination experiments were performed under aerobic conditions using TSB as a culture medium. For each set of experiments, both negative and positive controls were included. To determine the time leading to 50% of toxin removal (T50), the three strains were incubated in TSB (pH7) at 30 °C for 0, 3, 6, 8, 10, 12, 24 and 72 h in the presence of 1 µg/mL of ZEA. Bacterial growth was checked at each time point up to 72 h by plate counting onto TSA. To study the effect of temperature on ZEA removal, *B. subtilis* strains were incubated with ZEA (1 µg/mL) for 24 h in TSB (pH7) at 10, 20, 30 and 40 °C. The effect of medium pH on ZEA removal by the strains was evaluated by culturing each strain at 30 °C for 24 h in TSB adjusted to the following pH values: 3.0 ± 0.1, 5.0 ± 0.1, 7.0 ± 0.1 and 8.0 ± 0.1. The efficacy of the three strains in removing ZEA when added at different concentrations was also evaluated. The tubes containing TSB and the strains (6 Log CFU/mL) were incubated at 30 °C for 24 h, with 0.1, 0.5, 1.0, 5.0 and 10.0 µg/mL of the toxin.

### 2.6. Carbon Source Test

For carbon source tests, a basal mineral medium (MM) was prepared as indicated by Warner and Lokelma [62]. Bacterial cultures were grown overnight in 10 mL of a TSB medium in sterile disposable Sterilin™ Test Tubes up to ca. 9 Log CFU/mL and then centrifuged at 14,000 rpm for 5 min at 4 °C to collect the pellet. These latter tubes were then washed three times with 10 mL of sterile MM, and the bacterial suspension was finally resuspended in MM up to OD_600nm_ = 1. The inoculums were further diluted in sterile MM to reach a final concentration of ca. 6 Log CFU/mL. For the decontamination tests, 5 mL of each diluted culture was incubated with 1 µg/mL of each mycotoxin for 72 h at 30 °C under aerobic or anaerobic growth conditions. To demonstrate the viability and concentration of the inoculum, microbial counts at 0 and 72 h of incubation were obtained by plate counting onto TSA as described in Section 2.2. The carbon source test was also performed in anaerobic growth conditions using MMN as described by Nakano et al. [66].

### 2.7. Study of the Mechanism for ZEA Decontamination

#### 2.7.1. ZEA Desorption from Bacterial Cell Pellets

Multiple ZEA desorption studies were carried out to assess whether the ZEA decrease by *B. subtilis* strains is due to the adsorption of the toxin onto the bacterial cell wall or within the cells, rather than its metabolization. To this aim, bacterial strains were incubated for 24 h in TSB at 30 °C and pH7 in the presence of ZEA at 1 µg/mL. Bacterial cultures were centrifuged (20 min at 14,000 rpm), and supernatants were analyzed for residual mycotoxin content.

The first set of desorption experiments was performed to determine whether the toxin binds to the external cell walls. The pellets obtained above were washed twice with PBS and then resuspended in 1 mL of acetonitrile/water (90:10, *v*/*v*). The suspensions were incubated at 37 °C, 150 rpm for 1 h. After incubation, samples were centrifuged for 20 min at 14,000 rpm and supernatants were collected, dried under an air stream at ca. 50 °C, and then reconstituted with 500 µL of water/methanol (85:15, *v*/*v*) for UPLC-FLD analysis of the desorbed fraction of the toxin.

Further desorption studies allowed us to evaluate whether the toxin is entrapped into the cells (intracellular adsorption). To this end, the pellets washed twice with PBS were resuspended in 1 mL of acetonitrile/water (90:10, *v*/*v*) and then sonicated in an ice-bath using an ultrasonic homogenizer (Q500 sonicator, Qsonica Newtown, CT, USA) equipped with a 3.175 mm probe. The frequency was set to 20 KHz, 50% amplitude, and a 15 s ON/15 s OFF cycles (six times). After sonication, samples were centrifuged at 4 °C for 20 min at 14,000 rpm to remove cellular debris, and the supernatants (containing the toxin desorbed from the internal part of the cells) were recovered. Supernatants were dried under air stream at ca. 50 °C and reconstituted with 1 mL of water/methanol (85:15) for UPLC-FLD analysis of ZEA. A water/methanol (85:15, *v*/*v*) solution supplemented with 1.0 µg/mL of ZEA was used as a control.

#### 2.7.2. ZEA Decontamination by Culture Supernatants

To study the ability of extracellular enzymes of *B. subtilis* strains to remove ZEA, TSB was inoculated with each strain and incubated at 30 °C for 24 h with soft agitation (150 rpm). Cultures were centrifuged and 1 mL of each extracellular fraction was filtered (0.22 µm), supplemented with ZEA (1.0 µg/mL), and incubated in sterile screw-cap Eppendorf tubes at 30 °C for 24 and 72 h.

To verify if ZEA degradation of extracellular enzymes is an induced activity by the toxin, bacterial strains were cultured in TSB and the presence of the toxin as described in Section 2.3. After cultivation at 30 °C for 24 h, the cells were removed by centrifugation and filtration (0.22 µm). Each extracellular fraction (1 mL) was supplemented with ZEA (1 µg/mL) and incubated in a sterile screw cap Eppendorf tube at 30 °C for 24 and 72 h. In all cases, TSB supplemented with 1 µg/mL of ZEA was used as a reference, and the amount of ZEA removal by supernatants was quantified as detailed in Section 2.4.

#### 2.7.3. Effect of Heat- and Acid-Treated Bacteria on ZEA Removal

These experiments on dead biomass were performed according to El-Nezami et al. [67] with minor modifications. Bacteria cultures containing 9 Log CFU/mL were washed once with PBS, resuspended in 2 mL PBS and incubated in PBS for 1 h (viable bacteria), or boiled for 1 h (heat-treated bacteria). Acid-treated bacteria were prepared incubating 2 mL of the cell suspension with 2 mL of 2 M HCl for 1 h. After each of these treatments, the bacterial suspensions were centrifuged (14,000 rpm for 20 min at 4 °C) and the supernatants were removed. The bacterial pellets were resuspended in 2 mL of sterile PBS containing 1.0 µg/mL of ZEA, incubated at 30 °C for 60 min, and then centrifuged. The amount of ZEA removal was assessed by analyzing the supernatants by UPLC-FLD (Section 2.4). Bacterial viability in the recovered pellets was assessed by plating onto TSA as previously described. To assess the adsorption efficacy of viable heat- and acid-treated bacteria, desorption experiments were performed. Cell pellets were resuspended with 1 mL of acetonitrile/water (90:10, *v*/*v*), and then shaken at 37 °C for 60 min with soft agitation (150 rpm). After this incubation period, samples were centrifuged for 20 min at 14,000 rpm and supernatants were collected, dried under an air stream at ca. 50 °C and then reconstituted with 500 µL of water/methanol (85:15, *v*/*v*) for UPLC-FLD analysis. All assays were performed in triplicate, and positive and negative controls were included.

#### 2.7.4. Preliminary Enzymatic Hydrolysis Tests to Study the Mechanism of ZEA Removal

As a first attempt to study the mechanism of ZEA removal, enzymatic hydrolysis tests were performed in liquid media. This research aims to facilitate the future identification of putative ZEA conjugates/metabolites generated during the decontamination process by *B. subtilis* A5, B7 and C9. To this aim, 1 mL of the supernatants obtained after 24 h of decontamination trials in TSB (24 h-TSB) or MM (24 h-MM), was diluted with 5 mL of ammonium acetate buffer (0.1 M, pH 4.8) and incubated in a shaker (150 rpm) at 37 °C with 25 μL of the β-glucuronidase/arylsulfatase (30/60 U/mL) solution for 16 h. Then, the samples were adjusted to pH 7, cleaned using ZearalaTest™ WB immunoaffinity (IMA) columns, and analyzed by LC. In addition, β-glucuronidase and arylsulfatase were tested individually. Before testing, these enzymes were diluted to 30 and 60 U/mL, respectively. ZEA recovery experiments with each enzyme were performed as described above.

### 2.8. ZEA Decontamination in a Liquid Food Model

The liquid food model (LF) was prepared as described by Ragoubi et al. [29]. The growth of the three strains in LF was determined after 24 and 48 h using TSA as described above. To evaluate the efficacy of *B. subtilis* strains in removing ZEA, sterilized LF containing 1 μg/mL of toxin was inoculated with ca. 6 Log CFU/mL of each strain as a single culture or as a pool of all strains. Inoculated LF in the absence of the mycotoxin, and un-inoculated LF supplemented or not with the toxin were included as controls. The pH of LF was measured in diluted samples by using a pH meter (Φ 10 pH Meter, Beckman Instruments, Inc., Fullerton, CA, USA) at the beginning and the end of the incubation. Test samples and controls were incubated for 24 or 48 h at 30 °C under aerobiosis and static conditions. To measure ZEA removal, inoculated cultures in LF were analyzed for residual ZEA according to the validated method described by Ragoubi et al. [29]. The decrease in ZEA concentration was expressed as a percentage (%) and calculated according to the following formula: % ZEA removed = (C_t0_ − C_24/48h_/C_t0h_) × 100; where C_t0_ is the concentration of ZEA determined in the positive control cultures (uninoculated LF spiked by the toxin) and C_24/48h_ represents the mycotoxin concentration determined after 24 or 48 h of incubation with *B. subtilis* strains.

### 2.9. Statistical Analysis

Mycotoxin removal was calculated by comparison of the results of individual experiments with the corresponding control positive samples. As reported by Ragoubi et al. [29] the amount of removed mycotoxin was calculated as the difference between the amount of mycotoxin in the supernatant of the positive samples with no bacterial strain and the amount found in the supernatant of the experimental samples containing the strains. This amount was related then to the quantity present in the supernatant of the positive samples and expressed in percentage. According to Ragoubi et al. [29], the quantity of ZEA desorbed from cell pellets was measured by comparing the amount of toxin desorbed from the cell pellets to the amount removed by the bacteria and expressed in percentage. All results are presented as means of triplicate independent experiments, and their standard deviations. Data were analyzed by SigmaPlot^®^ (version 12.0). To find the significant differences among means, one-way and two-way analysis of variance (ANOVA) and the Tukey test (for post-hoc comparisons) were applied. In each case, the significance level was set at 0.05. In addition, a normality test (Shapiro–Wilk) and the Equal Variance test were used to check, respectively, the normally distributed population and constant variance assumption.

## 3. Results and Discussion

### 3.1. Mycotoxins Removal by B. subtilis

**Figure 1:**
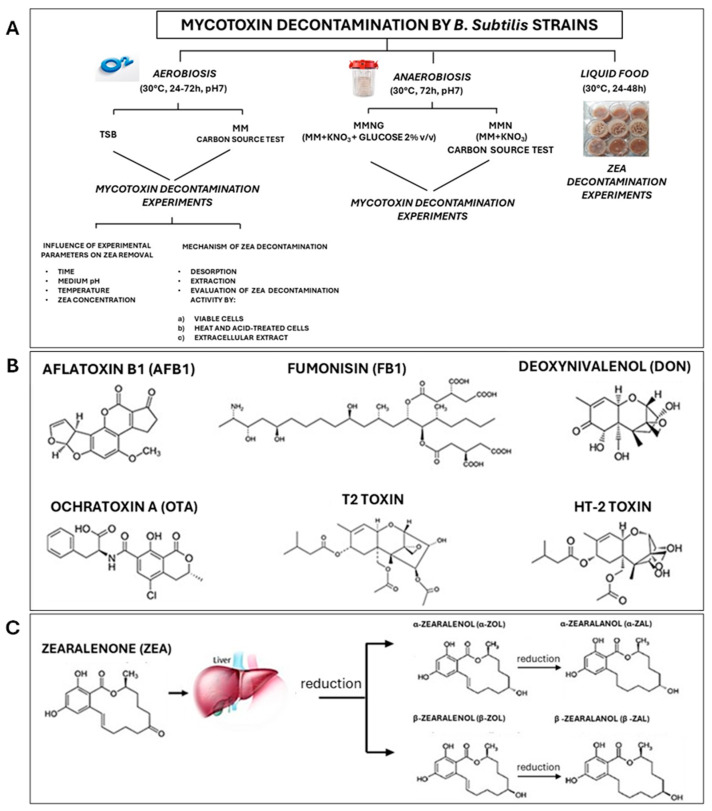
Mycotoxins decontamination by *B. subtilis* strains A5, B5, and C9. (**A**). Flowchart describing the activities performed to assess the efficacy of the *B. subtilis* strains to decontaminate mycotoxins under the different experimental conditions. TSB: Tryptic Soy Broth; MM: Minimal Medium; ZEA: zearalenone. (**B**). Mycotoxins tested in this study. (**C**). ZEA and its major metabolites.

The *B. subtilis* strains tested in this study were selected according to their genetic basis and safety. As stated by the Panel on Additives and Products or Substances used in Animal Feed (FEEDAP) of EFSA, the use of these *Bacillus* strains in animal nutrition is considered safe for all animal species, consumers, and the environment as the identity of the strains has been clearly established and they did not show acquired resistance to antibiotics of human and veterinary importance [46]. Therefore, the strains are recognized by EFSA as eligible for the Qualified Presumption of Safety (QPS) approach [46]. In addition, the whole-genome sequencing of the strains highlighted various genes potentially involved in mycotoxins degradation, such as CotA, coding a laccase involved in ZEN and AFB1 degradation; eFeB coding a peroxidase (BsDyp) involved in ZEA, AFB1 and DON degradation; BacC, coding an oxydoreductase (AFB1 degradation), DacA and DacB, coding a carboxypeptidase (OTA degradation) [68,69]. Considering these characteristics, the strains were evaluated for their ability to remove multiple mycotoxins (AFB1, ZEA, FB1, DON, OTA, T-2, and HT-2) under various environmental conditions and tested in a food model (Figure 1).

Many studies have attempted to investigate the application of various species of bacteria from the genus *Bacillus* to detoxify mycotoxins. However, only a few of them have demonstrated the efficacy of this genus against mycotoxins of primary concern, focusing mainly on some mycotoxins such as ZEA and AFB1. To our knowledge, no in vitro study has verified the effectiveness of *B. subtilis* in removing mycotoxins under anaerobic conditions and in a minimal medium containing the toxin as the sole carbon source. Furthermore, no study to date has examined the effectiveness of this species to detoxify ZEA in a liquid food model.

#### 3.1.1. Mycotoxins Removal Under Aerobic Growth Conditions

**Figure 2:**
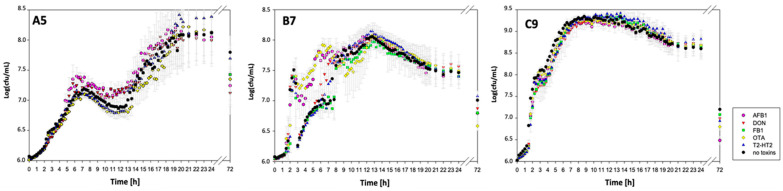
Kinetic growth curves of *B. subtilis* strains A5, B5, and C9 in TSB in the presence and absence of each mycotoxin. The graphs represent the growth (as Log CFU/mL) of the three strains (A5, B7, and C9) in TSB at 30 °C, pH 7 over a period of 24 h. Single point measurements taken after 72 h of incubation are also included. Different colors for data points represent the growth of *B. subtilis* strains in TSB supplemented with 1 µg/mL of each mycotoxin. Each data point represents the average of replicates (*n* = 9) with error bars indicating the standard deviation.

The efficacy of A5, B7, and C9 was evaluated in aerobic conditions, incubating each strain for 24 and 72 h in TSB supplemented with 1 µg/mL of each mycotoxin at 30 °C and pH 7. All *B. subtilis* strains were inoculated at 6.32 ± 0.21 Log CFU/mL, and bacterial growth in the presence and absence of each mycotoxin was followed up to 72 h after incubation. As shown in Figure 2, the maximum growth of A5 (8 Log CFU/mL) and C9 (9 Log CFU/mL) was observed after 20 and 12 h, respectively, while the strain B7 reached 8 Log CFU/mL after 12 h. However, after 72 h, the growth of the three strains decreased up to 7 Log CFU/mL regardless of the presence or absence of toxins. These results suggest that all the mycotoxins tested did not interfere with the growth of the strains (Figure 2). In addition, no effect on bacterial growth related to the presence of the acetonitrile used to dissolve mycotoxin standards (0.1% *v*/*v*) was recorded. The LC analysis of positive controls confirmed the stability of all mycotoxins during the 72 h of the experiment. Indeed, the amounts of AFB1, ZEA, FB1, DON, OTA, T-2 and HT-2 measured in sterile TSB remained unchanged throughout the experimental period (*p* < 0.05). Mycotoxins removal data (expressed as percentages and calculated as described in Section 2.8) under the above conditions are reported in Table 1. When cultured in TSB and aerobic conditions for 24 h, none of the three strains were effective in removing AFB1, FB1, DON, OTA, T-2, and HT-2 (removal levels < 20%). The same results were recorded with a longer incubation time (72 h). Notably, after 24 h, C9 exhibited a 38% removal in FB1, but this removal dropped to 9% after 72 h.

The literature provides some studies revealing the in vitro efficacy of *B. subtilis* in detoxifying mycotoxins focusing mainly on ZEA and AFB1 [9,56,70,71,72,73,74,75,76,77,78,79,80,81]. As can easily be inferred, mycotoxin degradation can occur in different ways depending on many factors, such as the species and strain used, the growth medium, the incubation time, the concentration of the bacteria cells, and the medium pH [56,74]. However, the efficacy of *B. subtilis* towards mycotoxins such as DON, OTA, FB1, T2 and HT-2 has been poorly investigated [82,83,84].

Although a significant mycotoxin removal activity was not observed for AFB1, FB1, OTA, DON, T-2, and HT-2, all *B. subtilis* strains removed ZEA, albeit at different levels depending on the strain. After 24 h of incubation in aerobic conditions, the most efficient strains were A5 and C9, which removed ZEA almost completely (>91%). ZEA removal by strain B7 was around 54%. After 72 h of incubation, the efficacy of A5 and C9 strains did not change, whereas for B7, it increased up to 65%, while bacterial cell load dropped to ca. 7 Log CFU/mL due to natural cell death (Figure 2).

Despite numerous publications, little is known about the removal activity of *B. subtilis* on ZEA analogues (α- and β-ZOL, α- and β-ZAL and ZAL), which are known to act as potential endocrine-disrupting chemicals (EDCs) by altering hormone production (Figure 1) [55,85,86,87]. Thus, the efforts of this work were also focused on evaluating the efficacy of the strains in removing the major ZEA analogues (α- and β-ZOL, α- and β-ZAL, and ZAL), some of which have higher estrogenic activity than ZEA. As shown in Table 1, after 24 h of incubation (aerobic conditions), all strains also removed ZEA when it was combined with the analogues (ZEA_mix_). The strains A5 and C9 removed the ZEA in the mix by more than 93%, whereas B7 removed the toxin by 74%.

Notably, for the first time, *B. subtilis* strains were found also able to remove ZEA congeners (α- and β-ZOL, α- and β-ZAL and ZAL). Strains A5 and C9 showed similar efficacy in removing ZEA analogues with a higher efficacy toward α- and β-ZOL (>94%). The strain B7 showed higher efficacy towards β-ZOL (96%) than α-ZOL (87%). Generally, the removal activity of the three strains was lower for α-ZAL, β-ZAL, and ZAL, being in the ranges 69–71% for A5, 69–76% for C9, and 41–48% for B7 (Table 1). The decontamination pattern observed in 24 h for ZEA and its analogues was confirmed after 72 h of incubation.

#### 3.1.2. Mycotoxins Removal Under Anaerobic Growth Conditions

Considering the results of this preliminary study, mycotoxin decontamination in anoxic growth conditions was assessed after 72 h of incubation in the MMNG growth medium at 37 °C and pH 7. To our knowledge, this is the first time that *B. subtilis* strains have been assessed for their ability to decontaminate in vitro mycotoxins in anaerobic growth conditions. The growth of the strains observed after 72 h of incubation in anaerobic conditions did not differ substantially from that observed in aerobiosis. Under these experimental conditions, none of the three strains was able to remove AFB1, FB1, DON, OTA, T-2 and HT-2 after 72 h of incubation. All of them showed a high decontamination efficacy towards ZEA and its metabolites. In particular, *B. subtilis* A5 was found to be the most effective among those tested, followed by C9 and B7 (Table 1). A5 and C9 strains decreased >96% ZEA when the toxin was tested alone or pooled with its analogues. Both strains could also remove ZEA analogues when cultured in the absence of oxygen. Specifically, ZEA and all its analogues (α- and β-ZOL, α- and β-ZAL, and ZAL) were undetectable in the liquid fraction of A5 bacterial cultures, whereas C9 removed α- and β-ZOL by 100%, and ZAL, α- and β-ZAL by 69–87%. Finally, strain B7 removed ZEA by 80 and 44% when tested alone or in the pool, respectively, and removed the other metabolites by 20–57%, with α-ZOL representing the most removed toxin. Interestingly, in all cases, the disappearance of ZEA in the medium by the three strains was not accompanied by the production of its metabolites. However, the strains can remove these metabolites as well.

Mycotoxin removal was also evaluated by culturing the pool of the strains A5, B7 and C9. After 24 h, the cell load reached 9.2 ± 0.2 Log CFU/mL confirming the growth of the strains. After 72 h, the cell dropped to 7.03 ± 0.3 Log CFU/mL. As observed for the single strains, after 72 h of incubation in TSB (30 °C, pH7), the pool was unable to decrease AFB1, FB1, DON, OTA, T-2 and HT-2, but it was still effective in removing ZEA (100%). All these results suggest that the pooling of the *B. subtilis* strains did not improve their efficacy in removing mycotoxins other than ZEA under optimal growth conditions.

### 3.2. Effect of Incubation Time, Temperature, pH, and Toxin Concentration on ZEA Removal by B. subtilis

Microorganisms used in manufacturing industrial biotechnological products have their own optimum pH, incubation time and temperature range. All these factors can influence the microbial degradation of mycotoxins [88]. Thus, the study explored how the growth of *B. subtilis* strains and the removal of ZEA were influenced by variations medium pH, temperature, and toxin concentration. Identifying optimal growth conditions can provide valuable insights for formulating an effective strategy to control ZEA.

**Figure 3:**
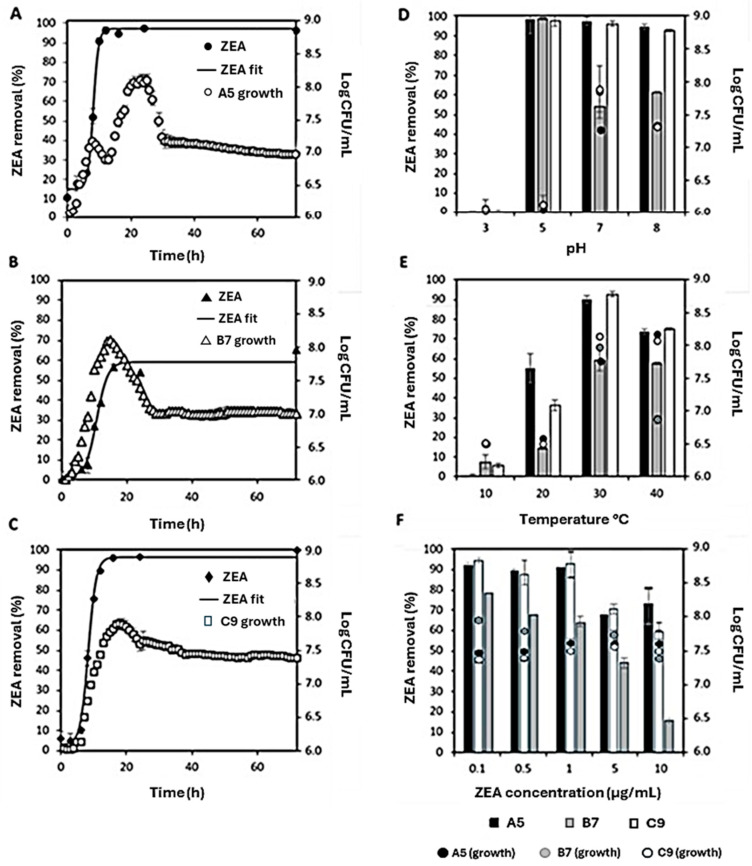
Effect of incubation time, temperature, pH, and toxin concentration on ZEA removal activity by *B. subtilis* strains. ZEA decontamination experiments were performed under aerobic growth conditions incubating A5 (**A**), B7 (**B**), and C9 (**C**) strains for 72 h at 30 °C and pH7 in the presence of 1 µg/mL of the toxin. The effect of medium pH (**D**), temperature (**E**), and ZEA concentration (**F**) on the decontamination activity of the strains were assayed in aerobic conditions, incubating each strain in TSB for 24 h. The growth of the strains (Log CFU/mL) over 72 h of incubation is also shown. Data represent the means and error bars are the standard deviations of three independent experiments.

Decontamination experiments were performed by culturing the three strains in TSB under aerobic conditions. The results of this study are shown in Figure 3.

#### 3.2.1. Effect of Incubation Time

Figure 3A–C refers to the effect of incubation time on ZEA removal by A5, B7 and C9 strains. The growth curve of these latter is also shown. For A5 and C9, a 50% removal of the initial toxin concentration (T_50_) was measured after 7.9 h and 8.4 h of incubation, respectively. The T_50_ calculated for B7 was 12.1 h. The time to reach 90% of ZEA removal was 9.9 h for A5 and 8.7 h for C9. Maximum ZEA decrease (97%) for A5 was achieved when the strains reached ca. 7 Log CFU/mL (14.3 h); after 72 h of incubation, ZEA removal by A5 did not change (Figure 3A). Maximum decrease in ZEA by C9 (96%) was reached when the strain reached ca. 8 Log CFU/mL (16 h), and it was kept constant until 72 h of incubation (Figure 3C). Maximum ZEA removal by B7 (59%) calculated from the curve fitting, was reached after 20 h of incubation, although experimental maximum removal (65%) was reached after 72 h of incubation. The maximum growth for B7 strain (8.1 Log CFU/mL) was reached after 13 h (Figure 3B). These results clearly show that strain A5 is the most effective among the strains tested herein, since it allowed a high ZEA removal (97%) even when reaching 7 Log CFU/mL after 14 h. In contrast, the high ZEA removal by C9 was obtained after 16 h when the concentration of the strain was 8 Log CFU/mL. It is evident from the results that the three strains herein tested, despite belonging to the same species, show different patterns of ZEA removal.

Although the strains examined in this study showed a growth rate as rapid as other *B. subtilis* strains known for their ability to decontaminate ZEA, they were able to remove the toxin in a shorter time. This feature is crucial for strains that should perform their action also in the gastrointestinal tract of animals. ZEA is quickly absorbed in the small intestine after ingestion, therefore ZEA detoxification must occur quickly after feeding.

#### 3.2.2. Effect of Medium pH

The incubation of strains in environments with different pH could impact the degradation rate of ZEA [88]. In addition, the pH found in the gastrointestinal tract of animals might be unfavorable for the proliferation of certain microorganisms and/or their enzymes. Therefore, to assess the effect of medium pH on ZEA removal by A5, B7 and C9 strains, bacterial cultures were grown in TSB at 30 °C for 24 h, with initial pH levels ranging from acidic (pH 3) to alkaline (pH 8). After 24 h of incubation, the pH values of bacterial cultures prepared at pH 3, 5 and 7 did not change significantly, whereas the pH values of bacterial cultures initially set at pH 8, decreased to pH 6.8. Therefore, the growth of *Bacillus* strains did not alter the pH of the medium, except when the latter was assayed at pH 8. As shown in Figure 3D, none of the strains exhibited a significant ZEA removal at pH 3. Furthermore, in this condition, no detectable bacterial growth was observed. After 24 h of incubation, the microbial load of the strains cultured at pH values in the range of 5–8 ranged from 6 Log CFU/mL (pH5) to 7.8 Log CFU/mL (pH7). Despite the low growth observed at pH 5, ZEA removal by strains A5 and C9 was significantly high in a large range of pH values (5–8). In this range, both strains removed more than 90% of the toxin and behaved in the same way. The B7 strain showed a different pattern of ZEA removal depending on the pH of the culture medium. Maximum ZEA removal (98%) was obtained at pH 5, despite the low growth rate recorded at this pH of the medium. A significant decrease in ZEA removal was observed when the strain was cultured at pH 7 and 8 (*p* < 0.001). At pH 7 and 8, ZEA removal values were 55% and 63%, respectively (Figure 3D). The optimal pH for ZEA removal observed in all strains falls within the activity range reported in the literature for other strains of *B. subtilis*, specifically pH 5–8 [88]. This feature makes the assayed strains suitable for various practical applications, including removal of the toxin in the small intestine, where ZEA is released from contaminated food matrices.

#### 3.2.3. Effect of Temperature

The study also examined the ZEA decontamination activity by strains A5, C9, and B7 cultured in TSB (pH7) for 24 h over a temperature range of 10 to 40 °C (Figure 3E). Temperature has a significant impact on microorganisms, and extremes, whether too high or too low, can influence the growth of bacterial strains as well as their metabolic activities. ZEA removal percentages and bacterial load expressed as Log CFU/mL are shown in Figure 3E. ZEA removal exhibited an upward trend in correspondence with increasing temperatures, peaking at 30 °C. When incubated at 10 °C for 24 h, bacterial load values remained close to the initial inoculum, and ZEA removal values stayed below 10%. At 20 °C, the bacterial load averaged around 6.5 Log CFU/mL, and higher removal rates for strains A5 (55%) and C9 (36%) were observed. At 30 °C, optimal ZEA reduction was achieved (*p* < 0.05), with strains A5, C9, and B7 yielding 97, 98 and 54% of removal, respectively. At this temperature, A5, B7 and C9 bacterial load increased to 7.8, 8.0 and 8.2, respectively. An increase in the temperature up to 40 °C produced a significant decrease in toxin removal values (≤75%) by strains A5 and C9, whereas ZEA removal by strain B7 was still at the maximum level. At 40 °C, A5 and C9 bacterial load remained at approximately 8 Log CFU/mL, whereas the bacterial load of B7 dropped up to 6.8 Log CFU/mL (Figure 3E). In conclusion, the maximum ZEA removal by A5 and C9 in TSB at pH7 was observed when incubating the strains at 30° C. The optimal temperature for the ZEA removal activity by B7 was in the range of 30–40 °C. Overall, the strains tested in this work were effective as early as 30 ° C, unlike other *B. subtilis* strains reported in the literature that require a higher temperature to exert their ZEA decontamination ability [44,78,80].

#### 3.2.4. Effect of Toxin Concentration

Lastly, ZEA removal by strains A5, B7 and C9 was investigated by incubating the strains for 24 h at 30 °C with increasing ZEA concentrations ranging from 0.1 to 10 µg/mL (Figure 3F). As in previous tests, the ZEA decontamination activity by strains A5 and C9 did not differ substantially. High removal rates (>90%) were observed for A5 and C9 when testing ZEA concentrations ranging from 0.1 to 1 µg/mL, while significantly lower removals (around 70%) were noted at higher ZEA concentrations (≥5 µg/mL). The bacterial load of A5 and C9 did not significantly change as the concentration of ZEA increased, remaining ca. 7.5 Log CFU/mL. As observed for A5 and C9, B7 decontamination activity significantly decreased when ZEA concentration was ≥5 µg/mL. Indeed, ZEA removal by B7 ranged from 78 to 65% when the initial toxin concentration was ≤1 µg/mL and decreased up to 40 and 10% when ZEA initial concentration was set at 5 and 10 µg/mL, respectively (*p* < 0.001). The bacterial load of B7 did not show a substantial change as the concentration of ZEA increased, settling between 7.8 and 7.5 Log CFU/mL (Figure 3F). It is noteworthy that, even at high ZEA concentrations, the removal activity exerted by the three strains was not accompanied by the production of ZEA analogues, such as α- and β-ZOL, α- and β-ZAL, and ZAL, as observed by Lei et al. [44] with the ZEA-degrading *B. subtilis* ANSB01G.

### 3.3. Carbon Source Test

The carbon source test, also known as the carbon source utilization test (CSU) or carbon assimilation test, is a microbiological technique used to determine the ability of bacteria to utilize various carbon compounds as a source of energy and carbon for growth. The literature lacks information regarding the efficacy of *B. subtilis* strains in detoxifying mycotoxins under minimal growth conditions. Therefore, mycotoxins decontamination by *B. subtilis* A5, B7 and C9 was also studied in aerobic conditions using MM as a growth medium. The viable cell load determination (ca. 6.5 Log CFU/mL) confirmed that after 72 h of incubation in MM at 30 °C and pH 7, no significant growth was observed compared to the initial inoculum. The presence of each mycotoxin did not impact this finding. As shown in Table 2, when cultured in MM and aerobiosis, A5 and C9 strains showed high efficacy in removing ZEA alone or in combination with the analogues. The percentage of ZEA and its analogues removed by these strains was around 100%. B7 removed more than 97% of ZEA (tested alone or in the mix), α- and β-ZOL, whereas the percentage of α-ZAL, β-ZAL and ZAL removed was 85%, 80% and 81%, respectively. As observed in the removal tests in TSB, all strains also failed to remove AFB, FB1, DON, OTA, T-2, and HT-2 in MM. Additionally, a comparison of results obtained in MM with those from TSB showed that culturing the strains in MM does not affect the efficacy of A5 and C9 in decreasing the levels of ZEA, α-ZOL, and β-ZOL. However, both strains showed a higher removal efficacy for α- and β-ZAL, and ZAL when cultured in MM rather than TSB. Notably, strain B7 exhibited a more pronounced decontamination activity against ZEA and its analogues when cultivated in MM, being variable between 80% (β-ZAL) and 99% (ZEA). These results could be attributed to a metabolic shift in the strains when grown in a nutrient-deficient medium.

The efficacy of *B. subtilis* strains in removing mycotoxins from MM after 72 h of incubation at 30 °C and pH 7 was also assessed under anoxic conditions using MMN as the growth medium (Table 2). As previously observed in aerobiosis, after 72 h of incubation in MMN, A5, B7, and C9 showed similar cell counts to the initial inoculum (ca. 6.50 Log CFU/mL). Among the three strains tested for removing ZEA from MMN, C9 was the most effective. However, its efficacy was lower than the activity observed under aerobic conditions. Low removal values were also observed for the other toxins with α- and β-ZOL representing the most decreased ones (40–50% removal). Under anerobic conditions, none of the strains removed AFB1, FB1, DON, OTA, T-2, or HT-2 (Table 2).

### 3.4. ZEA Removal by B. subtilis Strains: Insights into the Mechanism of Action

#### 3.4.1. ZEA Desorption Experiments

**Figure 4:**
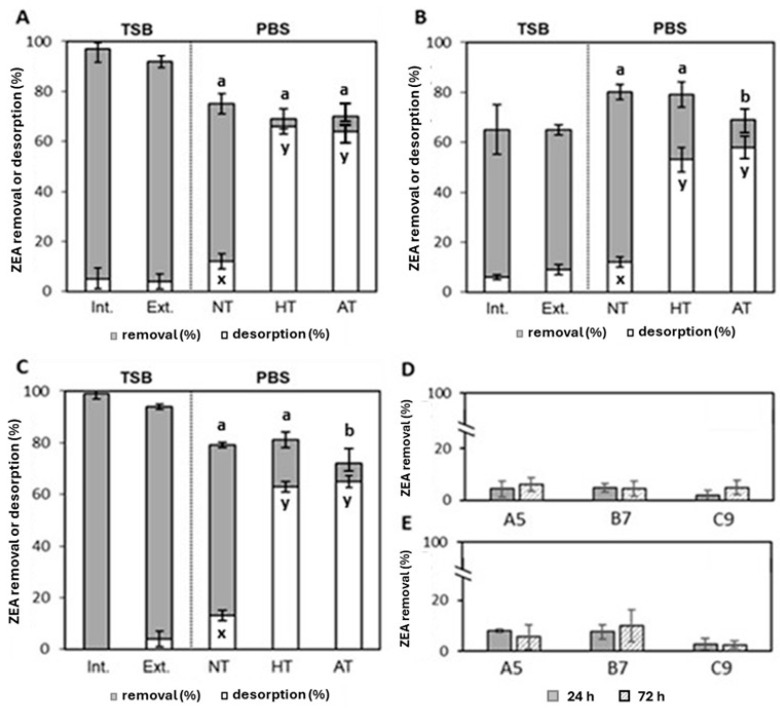
Percentages of ZEA removal and ZEA desorption from cell pellets of *B. subtilis* A5 (**A**), B7 (**B**) and C9 (**C**) obtained after decontamination trials performed in TSB or PBS. Decontamination trials in PBS were performed by incubating viable cell pellets (NT), heat-treated cell pellets (HT), and acid-treated cell pellets (AT) with the toxin. Red. (%) = percentage of ZEA removal; Des. (%) = percentage of ZEA desorption; Int. (%) = percentage of ZEA desorbed from cell pellets after sonication Ext. (%) = percentage of ZEA desorbed from cell pellets without sonication. For each strain, the ZEA removal values ^(a–b)^ and ZEA desorption values ^(x–y)^ were compared to assess significant differences among viable, heat- and acid-treated cells. (One-way ANOVA, Tukey test with *p* = 0.05 representing significance.) (**D**): ZEA removal by supernatants of bacterial strains cultured in TSB spiked with 1 µg/mL of ZEA. (**E**): ZEA removal by supernatants of bacterial strains cultured in TSB and pre-exposed to the toxin (1 µg/mL). All values represent the mean ± standard deviation of three independent experiments.

Desorption studies are a useful technique to determine whether the observed removal of ZEA by *B. subtilis* strains is due to the adsorption of toxin onto the bacterial cell walls rather than its actual biochemical removal. To this aim, bacterial strains were incubated with a known ZEA concentration to allow for adsorption onto the bacterial cell walls. After a defined incubation period, bacterial cells were removed from the solution. Then, a desorption agent was used to release the adsorbed fraction of the toxin from the bacterial walls. The concentration of ZEA in the supernatant after desorption was measured and compared to the initial concentration of ZEA to assess the amount that was adsorbed and later desorbed. If a significant amount of ZEA is adsorbed onto the bacterial cells, and desorption reveals that the majority of ZEA can be recovered, it suggests that adsorption is a significant factor in the removal observed. To gain a deeper understanding of the mechanism by which *B. subtilis* strains remove ZEA, multiple desorption experiments were performed using a 90:10 acetonitrile/water (*v*/*v*) mixture as the desorption agent, following the protocol outlined by Ragoubi et al. [29]. In the first set of desorption experiments, ZEA was not recovered from the cell pellets collected after 24 h of aerobic decontamination trials in TSB (containing 1 µg/mL of the toxin) at 30 °C and pH 7 (as in the Section 3.1.1). As illustrated in Figure 4A–C, ZEA desorption values for all three *Bacillus* strains were below 10%. This indicates that the removal of ZEA by these strains likely does not occur through adsorption onto the bacterial cell wall. Further studies were then performed to assess whether ZEA removal was due to an entrapment of the toxin into the bacterial cells (intracellular adsorption) rather than its metabolization. To this scope, the cell pellets obtained after aerobic decontamination experiments as above (24 h incubation time in TSB, at 30° and pH7) were sonicated in acetonitrile/water (90:10, *v*/*v*) and analyzed to assess the amount of toxin desorbed from the inner part of bacterial cells. As summarized in Figure 4A–C, ZEA extracted from the cell pellets after sonication was <6%. The major ZEA metabolites (α- and β-ZOL, α- and β-ZAL, and ZAL) were undetectable either in the supernatants or in the sonicated cell suspensions. These results suggest that a metabolism rather than an intracellular or extracellular binding can explain the removal of ZEA from TSB by *B. subtilis* strains.

Figure 4A–C also shows the results of ZEA removal and desorption from viable, heat- and acid-treated bacterial cells cultured in phosphate saline buffer (PBS). The results in Figure 4A–C indicate that viable cells of strains C9 and B7 exhibited marginally higher effectiveness, achieving approximately 80% ZEA removal, compared to strain A5, which achieved around 75% removal (*p* = 0.003). Only a small amount (≤13%) of ZEA was recovered after desorption experiments using viable cell pellets from all strains. These results are consistent with our experiments, which demonstrated a high efficacy of all strains grown in MM without additional carbon sources, in decontaminating ZEA under aerobic conditions (Table 2). Comparing these findings with the results in Table 2, where the strains were cultured in MM for 72 h, viable cells suspended in PBS removed over 75% of the toxin within just 1 h (Figure 4A–C). These results suggest a more rapid metabolization process of the toxin when viable cells are cultured in a mineral medium containing the toxin as the sole carbon source. In contrast, when cultured in a complex medium like TSB, the strains took over 8 h to remove the toxin by 50% (Figure 3). In addition, both heat- and acid-treated cells exhibited a comparable decrease in toxin levels after 1 h of incubation in PBS (Figure 4A–C). ZEA removals yielded by the bacterial cells after the heat and acid treatments were 69 and 70% for A5, 79 and 69% for B7, and 81 and 72% for C9. The acid-treated cells from strains C9 and B7 displayed a slight decrease in ZEA removal effectiveness, compared to the respective viable and heat-treated cells (*p* < 0.01). Nevertheless, when desorption experiments were performed on cellular pellets derived from cells subjected to heat or acid treatment, a substantial portion of the toxin removed by these non-living cells was successfully recovered (Figure 4). The percentages of ZEA desorbed by heat- and acid-treated cells were 66 and 64% for A5, 63 and 65% for B7, and 53 and 58% for C9. In summary, the outcomes of these experiments suggest that the removal of ZEA levels by *B. subtilis* strains can occur with both viable and non-viable cells, albeit through different mechanisms. For acid- or heat-treated cells, adsorption appears to be the primary mechanism responsible for toxin removal. In contrast, when viable cells are cultured in liquid media, including those containing the toxin as a carbon source, the removal is likely driven by metabolization. This process may involve enzymatic activities that metabolize the toxin, which is inactivated in acid- or heat-treated cells. *B. subtilis* strains can indeed produce various enzymes, such as cellulases, proteases, and amylases [34], which, in addition to improving food/feed quality, could take part in the process of biotransformation and degradation of ZEA and its metabolites. These findings are supported by several studies that suggest the ZEA degradation activity by *B. subtilis* strains is likely attributed to enzymatic activity, either of cellular or extracellular origin [44,73,88,89,90,91,92,93]. Presumably, the ability of bacteria to bind the toxin to their cellular surface may be a prerequisite for its absorption into the cells, where metabolization may occur.

#### 3.4.2. ZEA Removal by Extracellular Fractions of *B. subtilis*

To confirm the previous hypothesis and assess the ability of extracellular fractions of *B. subtilis* strains to remove the toxin, culture supernatants were prepared from strains incubated in TSB under optimal growth conditions (without ZEA). These supernatants were filtered, spiked with 1.0 µg/mL of ZEA, and incubated for 24 and 72 h. Additionally, supernatants obtained from ZEA aerobic decontamination experiments were aliquoted, supplemented with 1.0 µg/mL of ZEA, and incubated for 24 and 72 h. Figure 4D,E shows the percentages of ZEA removal achieved with unconcentrated, cell-free extracellular extracts from *B. subtilis* liquid cultures. No significant decrease in ZEA levels was observed when treated with these extracellular fractions, regardless of whether they were pre-exposed to ZEA (Figure 4E) or not (Figure 4D). Moreover, there was no notable difference between the two treatments. ZEA removal was minimal in all instances with decreases consistently below 10%. The results of this study suggest that the primary mechanism for ZEA removal by *B. subtilis* strains involves enzymatic activity within the bacterial cells or associated with their cell walls. Enzymes secreted into the liquid medium (extracellular enzymes) do not significantly contribute to the detoxification process. To our knowledge, most *Bacillus* strains that exhibit degrading activity towards ZEA primarily utilize extracellular metabolites (enzymes) [15,90,91]. This understanding can guide future research on bioremediation of ZEA by *B. subtilis* strains and its practical applications. Focusing on the mechanisms of intracellular and cell wall-associated enzymatic activities may prove more fruitful.

#### 3.4.3. Enzymatic Treatment of Extracellular Supernatants of *B. subtilis*

To further investigate the fate of ZEA removed by strains A5, B7, and C9, we performed a preliminary study in which the 24 h supernatants obtained from ZEA decontamination experiments in TSB or MM (under aerobic conditions) were treated with an enzymatic preparation containing β-glucuronidase and arylsulfatase. It is well-known that infected plants or certain fungal microorganisms can metabolize ZEA primarily through the formation of glucose or sulfate conjugates, such as ZEA-14-O-β-glucoside, ZEA-16-O-β-glucoside, and ZEA-14-sulfate, which exhibit lower estrogenic activity compared to the original molecule [75]. The LC analysis of the 24 h TSB supernatants from the three bacterial strains, after treatment with the enzymatic preparation, showed that ZEA was almost completely recovered, with a recovery rate of 97–100%. To determine which of the two enzymes were involved in the release of the toxin, the experiment was repeated by incubating the 24 h TSB supernatants with either β-glucuronidase or arylsulfatase individually. As a result, treatment with β-glucuronidase did not recover the toxin, while treatment with arylsulfatase led to the complete recovery of ZEA. These findings suggest that ZEA removal by *B. subtilis* in TSB is a metabolic phenomenon potentially involving the formation of ZEA adducts with sulfate or containing sulfur-linkages.

However, these results were not confirmed when the 24 h MM supernatants were enzymatically treated with both enzymes. Unlike the 24 h supernatants in TSB, those obtained from 24 h decontamination experiments in MM and treated with the hydrolases did not recover the toxin, with ZEA recovery values below 20%. Given that MM did not contain a sulfate source, further ZEA removal experiments followed by enzymatic recovery treatments were performed by replacing MgCl_2_ in the MM formula with MgSO_4_. Notably, the results of ZEA removal were confirmed, showing over 95% toxin removal after 24 h of incubation under aerobic conditions in MM supplemented with MgSO_4_. However, enzymatic treatments of the corresponding 24 h supernatants did not recover the toxin. This enzymatic approach should help us identify the metabolites resulting from ZEA degradation or biotransformation by strains A5, B7, and C9. Moreover, the results suggest that different metabolic pathways may be involved in the disappearance of ZEA by *B. subtilis* strains depending on the culture medium, leading to the production of different metabolites or adducts by these microorganisms.

Although these biotransformation products have not yet been identified, this study, for the first time, revealed that ZEA removal by *B. subtilis* in TSB may be due to a metabolic process potentially involving the formation of ZEA adducts with sulfate or sulfur linkages. In contrast, when the bacteria are cultured under stringent conditions, such as in MM containing ZEA as the sole carbon source, the biotransformation of ZEA appears to be more efficient, likely leading to the production of different conjugates or breakdown products. Further in-depth investigation is required to identify these metabolites and assess their safety.

In recent years, a growing number of probiotic *Bacillus* strains (i.e., *B. subtilis*, *B. natto*, *B. pumilus* ES-21, *B. licheniformis* CK1 and ZOM-1, *B. cereus* BC7, *B. mojaventis*, *B. velezenis* ANSB01E) have been studied for their ability to remove ZEA by a biotransformation mechanism [44,70,72,77,78]. Some of these studies showed high efficiency in mitigating the toxic effects of ZEA through animal feeding experiments on weaned female piglets [44]. However, biotransformation products were not mentioned in most of these reports. To our knowledge, few studies address the fate of ZEA during the fermentation process by *B. subtilis*, with existing research mainly attributing its transformation to phosphorylation, resulting in the formation of ZEA-14-phosphate [75,89], and diglycosilation modification, leading to ZEA-diglucoside [93]. ZEA adducts with sulfate or molecules bearing sulfur groups, produced through the biotransformation of ZEA by *B. subtilis* strains, have not yet been reported in the literature.

### 3.5. Evaluation of ZEA Removal Activity by B. subtilis Strains in a Liquid Food Model

Considering the overall results of the study—namely, the high efficacy of *B. subtilis* strains A5, B7, and C9 in removing ZEA within 24 h in TSB and mineral media, under both aerobic and anaerobic conditions; their acid tolerance and ability to remove the toxin at pH 5; their performance across a broad temperature range (20–40 °C), and their capability to remove ZEA at high concentrations (up to 10 µg/mL), including its more estrogenic analogues (α- and β-ZOL, α- and β-ZAL, and ZAL)—further experiments were performed to evaluate their effectiveness in decontaminating ZEA in a LF model. Fermented LF is extensively utilized in the animal industry as it contributes to animal welfare benefits. Fermented LF has been demonstrated to enhance the growth efficiency of pigs, decreasing the occurrence of diseases caused by enteric pathogens [29].

**Figure 5:**
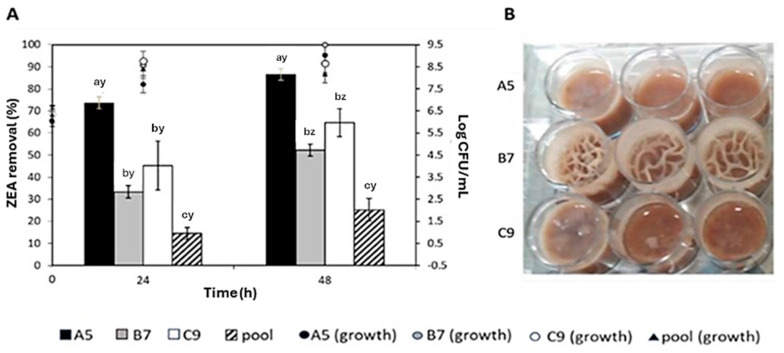
ZEA removal (%) in a liquid food model (LF) artificially contaminated with 1 µg/mL of the toxin and inoculated with *B. subtilis* strains A5, B7, and C9, singularly or as a pool (**A**). Inoculated LF was incubated for 24 and 48 h at 30 °C in aerobiosis. Bacterial load expressed as Log CFU/mL, determined after 24 and 48 h of incubation, is shown for A5 (growth), B7 (growth), C9 (growth), and the pooled strains (pool growth). ^a–c^ Different superscript letters indicate a significant difference of strains in removing ZEA from LF. ^y–z^ Different superscript letters indicate that the efficacy of strains was affected by incubation time (two-way ANOVA, Tukey test with *p* < 0.05 representing significance). Values represent mean ± standard deviation (error bars; *n* = 3). (**B**) appearance of A5, B7, and C9 growth in LF after 48 h of incubation. Three wells per strain are shown.

Decontamination experiments in LF were performed by incubating the strains (either alone or in combination) in LF containing 1 µg/mL of ZEA. According to Ragoubi et al. [29] the method developed to analyze ZEA in LF demonstrated high recovery rates (81–92%) and achieved within-day precision of ≤7%. The pH of LF measured immediately after its preparation was 5.71 ± 0.12 (on average), and no change was registered after 48 h of incubation (5.82 ± 0.17, on average). The strains A5 and B7 could grow up to ca. 9 Log CFU/mL after 48 h of incubation, whereas C9 reached ca. 8.5 Log CFU/mL (Figure 5A). Figure 5A shows the percentages of the ZEA removal in LF by the three strains after 24 and 48 h of incubation at 30 °C. A two-way ANOVA was used to compare the ZEA removal ability of the strains over 48 h of incubation. After 24 h, the ZEA removal from LF by strain A5 was significantly higher than C9 and B7 (*p* < 0.001). ZEA removal values recorded with the three strains decreased in the following order: A5 (74%) > C9 (43%) > B7 (33%). After a longer time of incubation (48 h), all strains showed a higher efficacy in removing the toxin (*p* < 0.001), with ZEA removal values of 87% for A5, 65% for C9, and 52% for B7. Therefore, a significant increase in the ZEA removal ability by *B. subtilis* strains can be achieved by extending the incubation time over 48 h. Longer incubation times were not evaluated as LF is generally consumed in less than 48 h. Furthermore, ZEA removal effectiveness in LF of the strains as a mix (pool) was also assessed as above. However, it was significantly lower than that observed for the single strains (*p* < 0.001). ZEA removal values yielded by the pool over 24 and 48 h were 15 and 25%, respectively (Figure 5A). Contrary to expectations based on individual strain performance, the pooled strains exhibited a diminished ZEA decontamination ability compared to their single counterparts. This finding underscores the necessity of developing tailored strategies when dealing with complex matrices such as liquid food.

## 4. Conclusions

The study provides a comprehensive exploration into the capabilities of *B. subtilis* strains A5, B7, and C9 in mitigating mycotoxin contamination within diverse environments, yielding multifaceted insights into their remediation potential.

The ability of *B. subtilis* strains to remove mycotoxins, tested herein, appears to be specific and toxin-dependent, as all three species examined demonstrated robust activity only against ZEA and its analogues, under aerobic and anaerobic growth conditions. All of them were ineffective against other major mycotoxins (AFB1, ZEA, FB1, DON, OTA, T-2 and HT-2). The high ZEA removal activity was observed in a large range of pH values (5–8), temperatures (20–40 °C), and at high toxin concentrations (up to 10 µg/mL). Interestingly, under restrictive growth conditions—simulated by replacing the nutrient-rich medium with a minimal one containing ZEA and/or its analogues as the sole carbon source—the ability to remove ZEA appears to be enhanced. These findings and mechanistic elucidation suggested that ZEA removal is a metabolic phenomenon (rather than merely a result of physical adsorption), involving enzymatic activities located within the bacterial cells or associated with their cell wall. The involvement of extracellular enzymes can be excluded. Interestingly, it has been observed that growth conditions and culture media can produce a shift in the metabolization/conjugation of the toxin, which can likely lead to the production of different metabolites/conjugates. In a rich medium, like TSB, the removal of ZEA can be ascribed to the production of conjugates containing sulfate or sulfur linkages. In a mineral medium where the toxin is the sole carbon source, its metabolism may result in the production of other metabolites or adducts, which will be identified and characterized for their properties and safety.

Extending our investigation into real-world scenarios, we explored the strains’ effectiveness in an LF model. The results revealed an intriguing outcome: strains A5 and B7 demonstrated robust proliferation, achieving significant increases in bacterial biomass and exhibiting enhanced efficacy in ZEA removal over a 48 h incubation period. In contrast, strain C9 showed a slightly lower bacterial loads but still maintained substantial efficacy.

In view of these results and the practical applications of *B. subtilis* strains A5, B7, and C9 in the food and feed industry, it should be noted that these strains are not genetically modified; their whole genome has been sequenced, revealing various genes that are potentially involved in mycotoxins degradation, and are recognized by EFSA as eligible for the QPS approach. Indeed, their use in animal nutrition is considered safe for all animal species, consumers and the environment. The findings of our study evidence the promising potential of these bacterial strains in mitigating ZEA contamination in food and feed commodities. ZEA, an estrogenic mycotoxin, not only compromises livestock productivity but also poses significant health risks to humans, leading to considerable economic losses. Traditional methods for reducing ZEA contamination—such as physical and chemical techniques—are often limited by high operational costs and the risk of nutrient degradation. In contrast, ZEA biotransformation through the application of these bacterial strains offers a sustainable and effective alternative for mitigating toxic impacts and minimizing food waste.

## Figures and Tables

**Table 1:** Mycotoxin removal (%) by *B. subtilis* A5, B7 and C9 strains under aerobic and anaerobic growth conditions. The decontamination activity of *Bacillus* strains was evaluated in aerobic conditions incubating the strains in TSB after 24 and 72 h at 30 °C and pH7. The decontamination activity of *Bacillus* strains under anoxic growth conditions was evaluated in MMNG after 72 h of incubation at 30 °C and pH7. Each mycotoxin was tested at 1 µg/mL. The values are mean ± sd of three independent replicates.

Mycotoxin	MYCOTOXIN REMOVAL (%, Mean ± sd, n = 3)								
	Aerobic Growth Conditions						Anaerobic Growth Conditions		
	24 h			72 h			72 h		
	A5	B7	C9	A5	B7	C9	A5	B7	C9
ZEA	96 ± 1 ^a^	54 ± 3 ^b^	91 ± 0 ^a^	97 ± 1 ^a^	65 ± 2 ^b^	97 ± 2 ^a^	98 ± 1 ^a^	80 ± 2 ^b^	96 ± 1 ^a^
AFB1	3 ± 1	3 ± 1	2 ± 1	3 ± 1	2 ± 1	3 ± 1	2 ± 1	1 ± 1	1 ± 1
FB1	5 ± 3 ^a^	13 ± 4 ^a^	38 ± 3 ^b^	5 ± 3 ^a^	19 ± 4 ^b^	9 ± 1 ^a^	3 ± 2	4 ± 1	2 ± 1
OTA	14 ± 5 ^a^	14 ± 1 ^a^	11 ± 5 ^a^	7 ± 3 ^a^	10 ± 5 ^a^	16 ± 5 ^a^	2 ± 0	2 ± 1	2 ± 1
DON	2 ± 3	1 ± 0	2 ± 2	4 ± 1	3 ± 2	0 ± 0	2 ± 1	2 ± 0	1 ± 2
T2	2 ± 1	2 ± 1	6 ± 3	7 ± 1	1 ± 1	4 ± 4	3 ± 1	2 ± 2	2 ± 1
H-T2	3 ± 1	1 ± 1	3 ± 2	6 ± 2	4 ± 2	5 ± 3	4 ± 1	3 ± 1	1 ± 1
ZEA_mix_	93 ± 1 ^a^	74 ± 1 ^b^	97 ± 0 ^c^	91 ± 1 ^a^	73 ± 1 ^b^	96 ± 2 ^c^	100 ± 0 ^a^	44 ± 1 ^b^	96 ± 1 ^c^
α-ZOL	94 ± 1 ^a^	87 ± 1 ^b^	98 ± 0 ^c^	93 ± 1 ^a^	91 ± 1 ^a^	99 ± 0 ^b^	100 ± 1 ^a^	57 ± 3 ^b^	100 ± 0 ^a^
β-ZOL	96 ± 1 ^a^	96 ± 1 ^a^	99 ± 0 ^b^	92 ± 3 ^a^	97 ± 2 ^a^	97 ± 0 ^a^	100 ± 1 ^a^	47 ± 4 ^b^	100 ± 1 ^a^
α-ZAL	69 ± 0 ^a^	41 ± 0 ^b^	69 ± 1 ^a^	72 ± 2 ^a^	45 ± 3 ^b^	73 ± 2 ^a^	100 ± 0 ^a^	33 ± 1 ^b^	79 ± 1 ^c^
β-ZAL	71 ± 0 ^a^	48 ± 3 ^b^	76 ± 0 ^c^	74 ± 1 ^a^	52 ± 2 ^b^	80 ± 2 ^c^	100 ± 1 ^a^	45 ± 2 ^b^	87 ± 1 ^c^
ZAL	71 ± 1 ^a^	46 ± 4 ^b^	76 ± 1 ^c^	73 ± 2 ^a^	48 ± 2 ^b^	78 ± 1 ^c^	100 ± 1 ^a^	20 ± 3 ^b^	69 ± 1 ^c^

AFB1 (aflatoxin B1), FB1 (fumonisin B1), ZEA (zearalenone), ZEA_mix_ (zearalenone + analogues), DON (deoxynivalenol), OTA (ochratoxin A), α-ZOL and β-ZOL (α- and β-zearalenols), α-ZAL and β-ZAL (α- and β-zearalanols), ZAL (zearalanone). Different superscript letters in the same row and for each incubation time indicate significant differences in the efficacy of the strains (one-way ANOVA, Tukey test with *p* < 0.05 representing significance).

**Table 2:** Carbon source test. Mycotoxin removal (%) by A5, B7 and C9 strains under aerobic and anaerobic conditions was obtained by incubating each strain in MM (aerobiosis) or MMN (anaerobiosis) for 72 h. Each mycotoxin was tested at 1 μg/mL. The values are mean ± sd of three independent replicates.

Mycotoxin	MYCOTOXIN REMOVAL (%, Mean ± sd, *n* = 3)					
	AEROBIOSIS			ANAEROBIOSIS		
	A5	B7	C9	A5	B7	C9
ZEA	100 ± 0 ^a^	99 ± 0 ^a^	99 ± 0 ^a^	23 ± 4 ^a^	20 ± 1 ^a^	80 ± 2 ^b^
AFB1	1 ± 1	1 ± 1	1 ± 1	2 ± 1	1 ± 1	1 ± 1
FB1	4 ± 2	1 ± 1	3 ± 1	4 ± 1	3 ± 2	4 ± 1
OTA	2 ± 0	3 ± 1	3 ± 1	2 ± 1	2 ± 2	1 ± 0
DON	1 ± 3	1 ± 0	1 ± 2	1 ± 1	1 ± 1	1 ± 0
T2	4 ± 1	4 ± 2	3 ± 1	5 ± 2	3 ± 1	3 ± 2
H-T2	4 ± 1	3 ± 1	1 ± 1	4 ± 1	3 ± 0	3 ± 1
ZEA_mix_	100 ± 0 ^a^	99 ± 1 ^a^	99 ± 2 ^a^	11 ± 0 ^a^	5 ± 1 ^b^	58 ± 2 ^c^
α-ZOL	100 ± 1 ^a^	98 ± 1 ^a^	99 ± 0 ^a^	1 ± 1 ^a^	3 ± 1 ^a^	50 ± 0 ^b^
β-ZOL	100 ± 1 ^a^	97 ± 0 ^b^	99 ± 2 ^a^	11 ± 1 ^a^	9 ± 2 ^a^	40 ± 3 ^b^
α-ZAL	99 ± 2 ^a^	85 ± 1 ^b^	97 ± 1 ^c^	1 ± 0 ^a^	2 ± 3 ^a^	19 ± 3 ^b^
β-ZAL	99 ± 1 ^a^	80 ± 2 ^b^	93 ± 1 ^c^	7 ± 1 ^a^	7 ± 2 ^a^	18 ± 2 ^b^
ZAL	99 ± 1 ^a^	81 ± 4 ^b^	96 ± 0 ^a^	2 ± 1 ^a^	3 ± 2 ^a^	5 ± 1 ^a^

AFB1 (aflatoxin B1), FB1 (fumonisin B1), ZEA (zearalenone), ZEA_mix_ (zearalenone + analogues), DON (deoxynivalenol), OTA (ochratoxin A), α-ZOL and β-ZOL (α- and β-zearalenols), α-ZAL and β-ZAL (α- and β-zearalanols), ZAL (zearalanone). Different superscript letters in the same row and for each growth condition indicate significant differences in the efficacy of the strains (one-way ANOVA, Tukey test with *p* < 0.05 representing significance).

## Data Availability

The original contributions presented in this study are included in the article. Further inquiries can be directed to the corresponding author.
